# Further characterisation of differences between TL and AB zebrafish (*Danio rerio*): Gene expression, physiology and behaviour at day 5 of the larval stage

**DOI:** 10.1371/journal.pone.0175420

**Published:** 2017-04-18

**Authors:** Ruud van den Bos, Wouter Mes, Pietro Galligani, Anthony Heil, Jan Zethof, Gert Flik, Marnix Gorissen

**Affiliations:** Department of Animal Ecology and Physiology, Faculty of Science, Radboud University, Nijmegen, the Netherlands; National Institutes of Health, UNITED STATES

## Abstract

Zebrafish (*Danio rerio*) have become popular as model organism in research. Many strains are readily available, which not only differ morphologically, but also genetically, physiologically and behaviourally. Here, we focus on the AB and Tupfel long-fin (TL) strain for which we have previously shown that adults differ in baseline hypothalamus-pituitary-interrenal (HPI)-axis activity (AB higher than TL) affecting inhibitory avoidance behaviour (absent in AB). To assess whether strain differences are already present in early life stages, we compared baseline HPI-axis related gene expression as well as cortisol levels, (neuro)development related as well as (innate) immune system related gene expression, and light-dark as well as startle behaviour in larvae 5 days post fertilisation. The data show that AB and TL larvae differ in baseline HPI-axis activity (AB higher than TL), expression of (neuro)development and immune system related genes (AB higher than TL), habituation to acoustic/vibrational stimuli (AB habituate faster than TL) and light-dark induced changes in motor behaviour (AB stronger than TL). Our data show that already in larval stages differences exist between zebrafish of the AB and TL strain confirming and extending data of earlier studies. To what extent the mutation in *connexin 41*.*8*, leading to spots rather than stripes in TL, but also (possibly) affecting eye, heart and brain function, is involved in the expression of (some of) these differences needs to be studied. These results emphasise that differences between strains need to be taken into account to enhance reproducibility both within, and between, laboratories.

## Introduction

Zebrafish (*Danio rerio*) have become popular as model organism in research [1]. Different strains, such as AB, Tupfel Long-Fin (TL), Tübingen (TU) and leopard, and their inter-crosses are being used. However, it is becoming increasingly clear that these strains differ not only morphologically, such as in fin size or skin patterns, but also genetically, physiologically and behaviourally, both in the adult and larval stages [2–12]. These differences may lead to differences in the outcome of behavioural tests (see [13] for inhibitory avoidance). Systematically phenotyping of zebrafish strains at different life-stages is therefore, among others, critical for enhancing reproducibility of experiments both within and between laboratories.

Recently, we [9] have shown that TL, but not AB, zebrafish show 24 h retention of exposure to an electric shock in an inhibitory avoidance task. This was suggested to be related to increased baseline hypothalamus-pituitary-interrenal (HPI) axis activity in AB over TL fish, as measured by whole-body cortisol levels and telencephalic mRNA levels of genes related to HPI-axis activity, including *corticotropin-releasing factor* (*crf*), *corticotropin-releasing factor binding protein* (*cr-bp*) and *glucocorticoid receptor alpha* (*gr-alpha*; *nr3c1α)*. Increased baseline HPI-axis activity may affect offspring as mothers deposit cortisol and glucocorticoid receptor (GR; both mRNA and protein) in egg yolk, which among others have effects on baseline HPI-axis activity, (neuro)development, (innate) immune function and motor activity in larvae [14–17]. The HPI-axis is functional in larvae from day 3 onwards, *i*.*e*. following hatching [18–20].

The aim of this study was to address whether differences in baseline HPI-axis related, (neuro)development related and (innate) immune system related gene expression as well as in baseline cortisol levels are present in larval stages of AB and TL, *i*.*e*. at 5 days post fertilisation. We also studied behavioural correlates (light-dark behaviour and startle behaviour) to assess functional consequences.

We assessed expression levels of HPI-axis genes (*crf*, *crf-bp*, *gr-alpha*, *mineralocorticoid receptor* (*mr; nr3c2*) and *glucocorticoid receptor beta* (*gr-beta*; *nr3c1β*) as well as whole-larvae cortisol [21,22]. In addition, we assessed expression levels of genes involved in (neuro)development: *proliferating cell nuclear antigen* (*pcna;* cell proliferation/cell cycle control [23,24]), *brain-derived neurotrophic factor* (*bdnf*; synaptic formation, neuronal connectivity, survival and migration of new neurons [24–26]), *neuronal differentiation factor 1* (*neurod1*; neuronal determination and differentiation; regulation of neuronal survival and maturation [24,27], *insulin-like growth factor 1* (*igf1*; growth, brain development, maturation and neuroplasticity [28,29]), *growth hormone 1* (*gh1*; synchronisation of somatic growth and energy metabolism [30,31]) and *cocaine- and amphetamine-regulated transcript 4* (*cart4*; energy homeostasis and neuroendocrine control [32,33]). Finally, we assessed expression levels of (innate) immune system related genes whose expression is enhanced under increased (baseline) levels of cortisol [15]: *immune-responsive gene 1-like* (*irg1l*), the macrophage marker *macrophage-expressed gene 1* (*mpeg1;* also called *mpeg1*.*1*), and the immune-regulatory gene *suppressor of cytokine signalling 3a* (*socs3a)*. We also included the paralogue of *mpeg1*, the macrophage marker *macrophage-expressed gene 1*.*2 (mpeg1*.*2)*, suggested to be regulated by *mpeg1* [34].

Thus far, it has been shown in direct comparative studies that AB and TL larvae differ in their motor response to sudden changes from both dark to light and light to dark [4,10,11]. The latter may (indirectly) reflect differences in baseline HPI-axis activity [35–37]. In addition, comparing data from different studies suggests that AB larvae habituate more rapidly to repeated exposure of acoustic/vibrational stimuli using a 1s inter-stimulus interval than TL larvae (TL: [38]; AB: [39]). Recently it has been shown that IGF1 may play a role in habituation in zebrafish larvae [40]. Thus, we included these paradigms for two reasons: (1) as differences between AB and TL larvae herein may be related to differences in HPI-axis activity and neuroplasticity, and (2) to assess whether our strains are comparable to those of other laboratories as differences may occur between laboratories while using the same strain [5,41].

## Materials and methods

### Breeding, embryos and larvae

In-house bred adult (> 6 months) zebrafish of the AB and Tupfel long-fin (TL) strains from the fish facilities of the Department of Animal Ecology and Physiology (Radboud University, Nijmegen, the Netherlands) were used for egg production. They were kept in recirculation systems (bio-filtered Nijmegen tapwater, ~28°C, pH 7.5–8, conductivity ~320 microSiemens/cm; Fleuren and Nooijen, Nederweert, the Netherlands) in 2-litre aquaria (approximately 30 fish of mixed sex) under a 14h:10h light-dark cycle (lights on from 09.00h to 23.00h) fed twice daily (at 09.00 h (*Artemia* sp. and Gemma Micro 300 (Skretting, Wincham, Northwich, Cheshire, UK)) and 15.00 h (Gemma Micro 300 (Skretting, Wincham, Northwich, Cheshire, UK)).

Breeding started at least one hour after the last feeding of zebrafish (> 16.00 h). Two males and one female of the AB or TL strain, were placed in a zebrafish breeding tank, separated by a partitioning wall, with water of ~28°C and an artificial plant on the side of the female. Fish were placed in the dark until the next morning. After turning on the lights at 09.00h, the fish were allowed to acclimatise for a few min. Then, water was changed for clean warm water of ~28°C, the partitioning wall was removed and tanks were placed at a slight angle for at least 30 min, such that the fish had the possibility to move into shallow water to spawn. When no eggs were produced, water was once more renewed (28°C) and tanks were again placed at a slight angle for at least 30 min.

Eggs were collected and transferred to Petri dishes (*n* = 50 per dish) filled with E3 medium (287 mg/l NaCl, 13 mg/l KCl, 48 mg/l CaCl_2_, 81 mg/l MgCl_2_, 3 ml 0.01% (w/v) methylene blue, in dH_2_O). Petri dishes were placed in an incubator set at 28°C. E3 medium was changed at day 0, day 1, day 4 and day 5; unfertilised eggs, dead eggs/embryos/larvae and chorions were removed. Eggs, embryos and larvae were kept under a 14h:10h light-dark period (lights on: 09.00h – 23.00h); light phase: 300–350 lux; dark phase; 0 lux.

All experiments were carried out in accordance with the Dutch Animals Act (http://wetten.overheid.nl/BWBR0003081/2014-12-18), the European guidelines for animal experiments (Directive 2010/63/EU; http://eur-lex.europa.eu/legal-content/NL/TXT/HTML/?uri=CELEX:32010L0063 and institutional regulations.

### Gene expression analysis

Larvae (5 days post fertilisation; dpf) were sampled between 09.00 h and 13.00 h. They were deeply anesthetized by placing them in 0.1% (v/v) 2-phenoxyethanol. To obtain sufficient material for analysis, two larvae were transferred to 2-ml Eppendorf tubes containing a plastic grinding ball; thus, one sample contained material of two larvae. Residual medium was removed with a pipette and samples were snap-frozen in liquid nitrogen and stored at -80°C until total RNA extraction. Total RNA content of each sample was isolated. This was done by homogenising the tissue with 400 μl Trizol reagent (Invitrogen, Carlsbad, USA) in a Grinding Mill (Retsch GmbH, Germany) for 20 s at 20 Hz. After homogenisation, samples were kept at room temperature for 5 min. Next, 80 μl chloroform was added and the solution was mixed by shaking for 15 s. Afterwards, samples were kept at room temperature for 2 min. The samples were centrifuged at 14,000 rpm for 10 min in a cooled centrifuge (4°C) and the aqueous phase of the samples was transferred to a new tube. To this phase, 200 μl isopropanol was added and this solution was mixed well by inversion of the tube. The solution was then stored at -20°C for 2 h and centrifuged afterwards for 15 min at 14,000 rpm in a cooled centrifuge (4°C). The supernatant was decanted and the pellet washed with 500 μl 75% ethanol and centrifuged 10 min at 14,000 rpm in a cooled centrifuge (4°C). The supernatant was decanted, after which the pellet was centrifuged for 5 s to remove all the remaining supernatant using a pipette. The pellet containing the RNA was air-dried for 10 min at room temperature and afterwards dissolved in 100 μl DEPC-treated dH_2_O. To this RNA solution, 10 μl 3M NaAc (pH 5.4) and 250 μl 100% ethanol were added. The solution was mixed by inverting the tube and samples were stored for 2 h at -20°C. Subsequently, the samples were centrifuged for 15 min at 14,000 rpm in a cooled centrifuge (4°C) and the supernatant was decanted and the pellet washed as described earlier. Finally, the RNA was dissolved in 15 μl DEPC-treated dH_2_O. The concentration and quality of RNA in each sample were measured using a nanodrop spectrometer at 260 nm wavelength (Nanodrop, Wilmington, DE, USA).

Isolated RNA was treated with DNase to remove any (genomic) DNA from the sample. 400 ng RNA was transferred into a PCR strip, and DEPC-treated dH_2_O was added to a volume of 8 μl. To this, 2 μl of DNase mix was added, containing 1 μl 10x DNase I reaction buffer and 1 μl (1 U/μl) amplification grade DNase I (both from Invitrogen, Carlsbad, USA). The resulting mix was incubated for 15 min at room temperature. Afterwards, 1 μl 25 mM EDTA was added to stop the DNase reaction and the reaction mix was incubated for 10 min at 65°C and put back on ice.

After the DNase treatment, samples were used to synthesize cDNA by the addition of 1 μl random primers (250 ng/μl), 1 μl 10 mM dNTP mix, 4 μl 5 x 1^st^ strand buffer, 1 μl 0.1 M DTT, 1 μl RNase inhibitor (10 U/μl), 0.5 μl Superscript II (reverse transcriptase) (200 U/μl) (all from Invitrogen, Carlsbad, USA) and 0.5 μl DEPC-treated dH_2_O. The resulting mix was incubated for 10 min at 25°C for annealing of the primers and then 50 min at 42°C for reverse transcription. Hereafter, enzymes were inactivated by incubating samples at 70°C for 15 min. Finally, 80 μl dH_2_O was added to dilute the samples five times for the qPCR reaction.

To measure the relative gene expression in each sample, real-time qPCR was carried out for each gene of interest. For each qPCR reaction, 16 μl PCR mix (containing 10 μl SYBR green mix (2x) (BioRad, Hercules, USA), 0.7 μl forward and reverse gene-specific primer (10 μM) and 4.6 μl H_2_O) was added to 4 μl of cDNA. The qPCR reaction (3 min 95°C, 40 cycles of 15 s 95°C and 1 min 60°C) was carried out, using a CFX 96 (BioRad, Hercules, USA) qPCR machine. Analysis of the data was carried out using a normalisation index of two reference genes (*viz*. *elongation factor alpha* (*elf1a*) and *ribosomal protein L13* (*rpl13*)) [42]. Primer sequences of genes of interest are shown in Table 1.

**Table 1:** Nucleotide sequences of the forward and reverse primers used for qPCR.

Gene	accession	forward primer	reverse primer	amplicon
Name	number	Sequence	sequence	length
*elf1a*	AY422992	CTGGAGGCCAGCTCAAACAT	TCAAGAAGAGTAGTACCGCTAGCATTAC	85
*rpl13*	NM_212784	TCTGGAGGACTGTAAGAGGTATGC	AGACGCACAATCTTGAGAGCAG	147
*crf*	BC164878	CGAGACATCCCAGTATCCAAAAAG	TCCAACAGACGCTGCGTTAA	59
*crf-bp*	NM_001003459	ACAATGATCTCAAGAGGTCCAT	CCACCAAGAAGCTCAACAAA	66
*mr*	NM_001100403	CTTCCAGGTTTCCGCAGTCTAC	GGAGGAGAGACACATCCAGGAAT	74
*grα*	EF436284.1	ACTCCATGCACGACTTGGTG	GCATTTCGGGAAACTCCACG	90
*grβ*	EF436285.1	GATGAACTACGAATGTCTTA	GCAACAGACAGCCAGACAGCTCACT	210
*bdnf*	NM_131595	AGAGCGGACGAATATCGCAG	GTTGGAACTTTACTGTCCAGTCG	110
*pcna*	AF140608.1	AAGGAGGATGAAGCGGTAACAAT	GTCTTGGACAGAGGAGTGGC	104
*neurod1*	AF036148.1	TCCCTACTCCTACCAGACGC	CAGTCTGTGAGGGTGGTGTC	135
*igf1*	NM_131825.2	GGCGCCTCGAGATGTATTGT	CCTCGGCTCGAGTTCTTCTG	160
*gh1*	NM_001020492.2	GCATCAGCGTGCTCATCAAG	TGAGACTGGTCTCCCCTACG	114
*cart4*	NM_001082932.1	GCTGAGGCACTCGATGAACT	GAAGAAAGTGTTGCAGGCGG	174
*socs3a*	NM_199950.1	GCAGGAAGACTGTGAACGGA	CAGTGGCTGGACAACTCCAT	192
*irg1l*	NM_001077607.1	TTTCAAGCGCTTTCCTGCAC	GAGAGTGGCACCCTAAGCAG	141
*mpeg1*.*1*	NM_212737.1	CCACAGAAAGTGAGCGGAGT	GGACTTGAACCCGTGCTGTA	196
*mpeg1*.*2*	NM_001020586.1	CACGGCGGGGCTTTATTCTA	GAGGTCAGGGAATACGGCTG	135

Genes: **stress-axis genes:** *corticotropin-releasing factor (crf)*, *crf-binding protein (crf-bp)*, *mineralocorticoid receptor (mr)*, *glucocorticoid receptor α (grα)* and *β (grβ)*; **‘(neuro)development related’ genes:** *proliferating cell nuclear antigen (pcna)*; *brain derived neurotrophic factor (bdnf)*; *neuronal differentiation factor 1* ***(****neurod1)*; *insulin growth-factor 1 (igf1)*; *growth hormone 1 (gh1*); *cocaine- and amphetamine-regulated transcript 4 (cart4)*; ‘(i**nnate) immune system related’ genes:** *suppressor of cytokine signalling 3a* ***(****socs3a****)*,** *immune-responsive gene 1-like (irg1l)*, *macrophage-expressed gene 1 (mpeg1*.*1*; also called *mpeg1*), *macrophage-expressed gene 1*.*2 (mpeg1*.*2)*; **reference genes**: *elongation factor 1α (elf1α)*; *ribosomal protein L13 (rpl13)*.

### Cortisol

Larvae (5 dpf) were sampled around 13.00 h. They were deeply anesthetized by placing them in 0.1% (v/v) 2-phenoxyethanol. To obtain sufficient material for analysis, ten larvae were transferred to 2-ml Eppendorf tubes; thus, one sample contained material of 10 larvae. Residual medium was removed with a pipette and samples were snap-frozen in liquid nitrogen and stored at -20°C. Cortisol was extracted from larvae using a HCl extraction [43]. Each sample was thawed on ice and 15 μl ice-cold 0.01M HCl was added to the sample in an Eppendorf cup. Larvae were manually homogenised with a plastic homogenization tool (Greiner-Bio-One, Frickenhausen, Germany). After homogenisation, another 15 μl 0.01M HCl was added to the sample and mixed well. Samples were centrifuged 10 min at 14,000 rpm (4°C). After centrifugation, supernatant was transferred into a new Eppendorf while the pellet (containing the homogenised tissue) was discarded. The supernatant was centrifuged twice for 10 min at 14,000 rpm and the supernatant was transferred into a new Eppendorf after each centrifugation step. Collected supernatant was stored at -20°C until the cortisol content was measured using a custom radioimmunoassay (RIA; [44]; see below).

For the RIA, 96-wells Micro-Assay-Plates (Greiner-Bio-one: 655094; White/μClear–high-binding) were used, to which 100 μl cortisol antibody (*Abcam*: *ab1949; Cortisol Antibody[xm210] monoclonal and IgG purified*; 1:2000 dilution) in coating buffer (50 mM NaHCO_3_, 50 mM Na_2_CO_3_, 0.02% NaN_3_; pH = 9.6) was added. A-specifics received 100 μl coating buffer only, without the antibody. The plates were incubated overnight at 4°C. The next day, the plates were washed 3 times with 200 μl Wash Buffer (100 mM Tris, 0.9% NaCl, 0.02% NaN_3_; pH 7.4) in each well. Afterwards, 100 μl Blocking buffer (0.25% Normal Calf Serum in Wash Buffer) was added to each well, the plates were sealed and incubated at 37°C for 1 h, after which the fluids were removed by decanting (without washing). Ten μl of sample was added (in duplicate) to designated wells. A-specifics and B_0_ received 10 μl Assay Buffer and the standards to which a known concentration cortisol (4, 8, 16, 32, 64, 128, 256, 512, 1024 and 2048 pg cortisol / 10 μl) were added in triplicate to designated wells. Next, to each well, 90 μl ^3^H-Cortisol tracer (1 μl ^3^H-Cortisol/10 ml Assay Buffer: 333 Bq per well) was added, after which the plates were sealed and incubated overnight at 4°C.

After incubation, wells were emptied by decanting and washed 3 times using 200 μl Wash Buffer. After washing, 200 μl scintillation solution (Optiphase hisafe-3 scintillation liquid’; PerkinElmer, USA) was added to each well and the plate was sealed. Each plate was placed into a β-counter (Microbeta Plus; Wallac/PerkinElmer, USA) to measure the amount of radioactivity in each well. The cortisol content of samples was then calculated using the standards values. Inter-assay coefficient variation was 12.5% and intra-assay coefficient variation was 2.5%. Cross-reactivity of the antibody with other relevant steroids (11-deoxycortisol, corticosterone, 11-deoxycorticosterone, progesterone, 17-hydroxyprogesterone, testosteron, oestradiol and oestriol) was not considered significant (< 1% at 50% cortisol saturation).

### Behavioural assays

Motor activity of 5 dpf larvae in response to light-dark conditions or acoustic/vibrational stimuli was analysed using a DanioVision system (Noldus B.V., Wageningen, the Netherlands) using 24-wells plates at a temperature of 28°C using a heating/cooling system (Noldus B.V., Wageningen, the Netherlands). Following the behavioural experiments larvae were euthanized by ice slurry exposure for at least 20 minutes.

### Dark-light-dark activity

Larvae (5 dpf) were transferred from Petri dishes to wells filled with 1 ml E3 medium. Runs (*n* = 24 AB or *n* = 24 TL) from different clutches of AB or TL were tested in a randomised way at different days between 13.00h and 18.00h as it has been shown that activity is most stable in the afternoon [45]. The protocol (modified from [4]) consisted of 20 min acclimation (with lid of the system open; room light: 500–650 lux), closing of the lid followed by alternating periods of 10 minutes dark, 10 minutes bright light (about 3000 lux) and 10 minutes dark. Based on literature [4,10,11] and preliminary analyses, variables of interest were: *distance moved* (mm) for general activity under dark and light conditions, change in *maximum velocity* (mm/s) for the change from dark to light (*maximum velocity* first 30 s light condition minus maximum velocity last 30s of dark condition) and change in *distance moved* (mm) for the change from light to dark (*distance moved* first 30 s dark condition minus *distance moved* from last 30 s of light condition).

#### Acoustic/vibrational startle

Larvae (5 dpf) were transferred from Petri dishes to wells filled with 1–1.5 ml E3 medium. Runs (*n* = 12–24 AB or *n* = 12–24 TL) from different clutches of AB or TL were tested in a randomised way at different days between 13.00h and 18.00h as it has been shown that activity is most stable in the afternoon [45]. The protocol (lights on) consisted of 10 min acclimation, followed by 10 acoustic/vibrational stimuli (DanioVision intensity setting 6) with a 20 s inter-stimulus interval (ISI), a 10 min pause followed by 30 acoustic/vibrational stimuli with a 1 s ISI. This protocol was based on earlier studies showing no habituation to repeated stimuli under 20 s ISI and habituation under 1 s ISI [38–40, 46]. Variable of interest to show the startle response was *maximum velocity* (mm/s) with 1 s intervals, since the startle response is a short burst of activity best captured by this parameter. When subjects did not show a clear response to the first stimulus (values lower than 15 mm/s) they were discarded from analysis.

### Statistical analysis

Strain comparison for gene expression levels and whole-body cortisol levels were compared using Student’s *t*-tests following testing for normal distribution; outliers were removed following Grubb’s outlier test (*p* ≤ 0.01). For light-dark motor activity and acoustic startle, two-way analysis of variance (ANOVA), Student’s *t*-tests or one-sample t-tests were used where appropriate. All statistical analyses were done using IBM SPSS version 23 for Windows (IBM, Armonk, NY, USA). In all cases, significance was accepted when *p* ≤ 0.05 (two-tailed), unless otherwise stated.

## Results

### Gene expression levels and cortisol

Table 2 shows gene expression levels of 5 dpf AB and TL larvae for genes related to the HPI-axis, (neuro)development and the (innate) immune system. All gene expression levels but those of *mr* and *gh1* differed between AB and TL larvae: gene expression levels of *crf*, *crfbp*, *gr-beta*, *bdnf*, *pcna*, *neurod1*, *cart4*, *igf1*, *soc3a*, *mpeg 1*.*1*, *mpeg 1*.*2* and *irg1l* of AB larvae were significantly higher than those of TL larvae, while those of *gr-alpha* were lower. Cortisol values of AB larvae were significantly (*t* = 4.944, df = 12, *p* ≤ 0.001) higher than of TL larvae (mean ± SEM): 0.164 ± 0.007 ng/ml/larva (*n* = 6 samples) *versus* 0.073 ± 0.015 ng/ml/larva (*n* = 8 samples).

**Table 2:** Mean (± SEM) relative expression normalised to *elf1a/rpl13* for genes of interest in 5 dpf larvae of AB and TL.

Gene	AB (*n* = 10)	TL (*n* = 10)	Statistics
*crf*	2.75 ± 0.25	2.00 ± 0.16	*t* = 2.566; df = 18; *p* ≤ 0.02
*crf-bp*	1.60 ± 0.09	1.07 ± 0.07	*t* = 4.546; df = 18; *p* ≤ 0.001
*mr*	3.22 ± 0.33	2.67 ± 0.12	
*gr-alpha*	0.66 ± 0.04	1.30 ± 0.11	*t* = -5.368; df = 18; *p* ≤ 0.001
*gr-beta*	0.78 ± 0.09	0.10 ± 0.03	*t* = 7.508; df = 18; *p* ≤ 0.001
*bdnf*	1.70 ± 0.07	0.58 ± 0.09	*t* = 9.735; df = 18; *p* ≤ 0.001
*pcna*	0.49 ± 0.03	0.14 ± 0.01	*t* = 11.689; df = 18; *p* ≤ 0.001
*neurod1*	1.33 ± 0.11	0.77 ± 0.09	*t* = 3.899; df = 18; *p* ≤ 0.001
*gh1*	0.10 ± 0.01	0.09 ± 0.01	
*cart4*	0.41 ± 0.04	0.12 ± 0.03	*t* = 5.457; df = 18; *p* ≤ 0.001
*igf1*	1.89 ± 0.21	0.83 ± 0.07	*t* = 4.693; df = 18; *p* ≤ 0.001
*socs3a*	0.07 ± 0.004	0.04 ± 0.006 (n = 9)	*t* = 4.175; df = 17; *p* ≤ 0.001
*mpeg1*.*1*	0.13 ± 0.02 (n = 9)	0.08 ± 0.008	*t* = 2.281; df = 17; *p* ≤ 0.04
*mpeg1*.*2*	0.50 ± 0.07	0.05 ± 0.01	*t* = 5.996; df = 18; *p* ≤ 0.001
*irg1l*	0.24 ± 0.05	0.03 ± 0.008 (n = 7)	*t* = 3.807; df = 15; *p* ≤ 0.002

Unless otherwise indicated *n* = 10 samples per strain (2 larvae/sample); Reasons for less than 10 samples: poor signal (AB: *mpeg1*.*1*; TL: *irg1l* (n = 2)) or outliers (Grubb’s test, *p*≤0.01; TL: *socs3a*; TL: *irg1l*); Statistics between AB and TL are shown (Student’s *t*-test).

#### Light-dark activity

**Fig 1:**
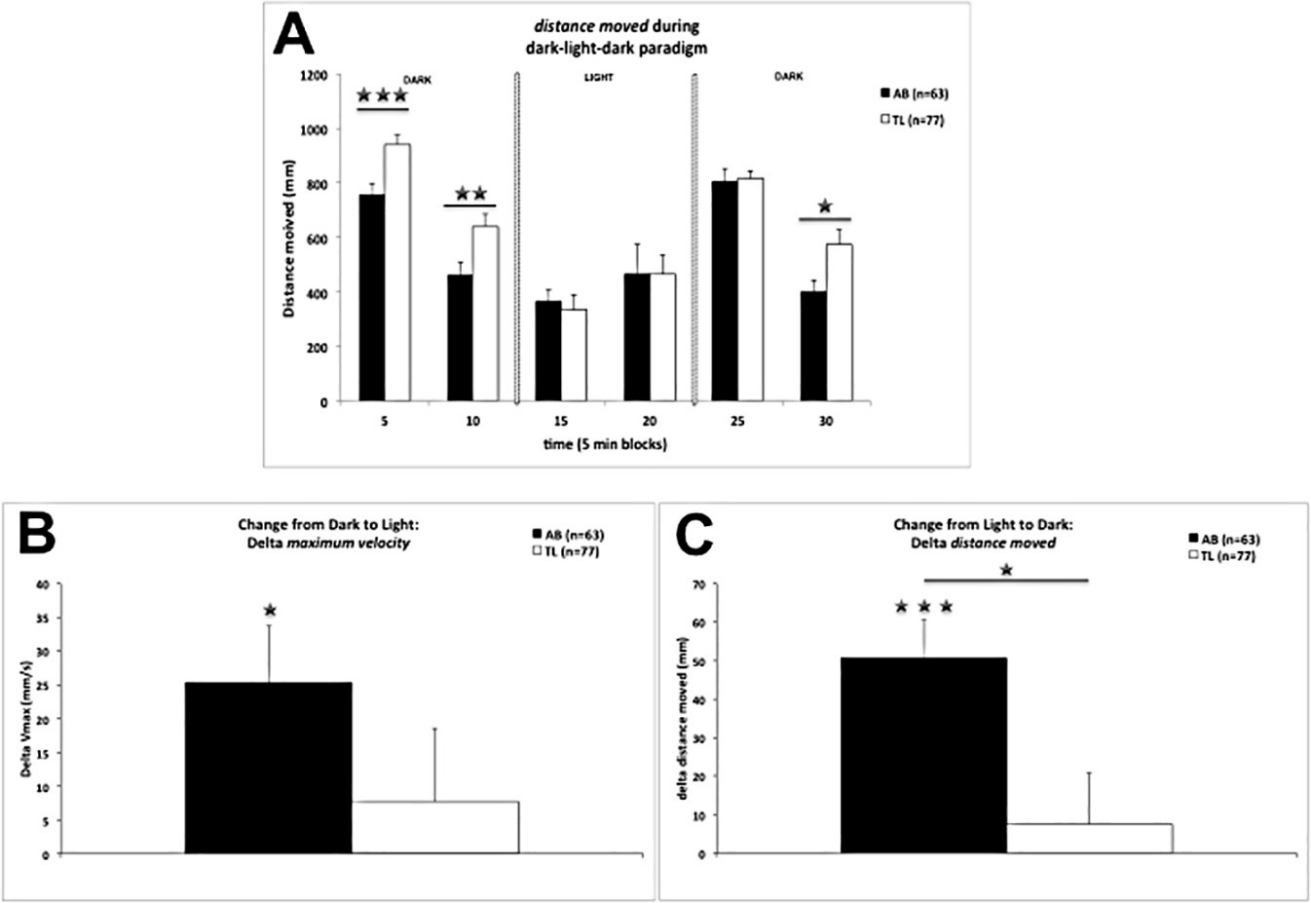
Dark (10 min)–light (10 min)–dark (10 min) paradigm for AB and TL larvae. **Panel A**: *Distance moved* (means + SEMs) for AB and TL larvae across the different light-dark periods (for statistics see text; between strains: Student’s *t*-test; *: *p ≤ 0*.*05*, **: *p ≤ 0*.*01*, ***: *p ≤ 0*.*001)*. **Panel B**: Change in *maximum velocity* (Δ of the first 30s of the light period and the last 30s of the dark period; means + SEMs) from dark to light for AB and TL larvae (statistics: values different from 0 (*i*.*e*. no change) per strain (one-sample *t*-test): *: *p* ≤ 0.05 (1-tailed values [10,11])). **Panel C:** Change in *distance moved* (Δ of the first 30s of the dark period and the last 30s of the light period; means + SEMs) from light to dark for AB and TL larvae (statistics: values different from 0 (*i*.*e*. no change) per strain (one-sample *t*-test): ***: *p* ≤ 0.001 (1-tailed values [10,11]); between strains: Student’s *t*-test; *: *p* ≤ 0.05).

In Fig 1A overall motor activity (*distance moved*) is shown of 5 dpf AB and TL larvae in the dark-light-dark paradigm. While AB and TL larvae changed activity across time blocks (repeated-measures two-way ANOVA (factors: strain and time blocks; time blocks: *F*(5,690) = 35.763, *p* ≤ 0.001), they did not so in the same way (time block × strain: *F*(5,690) = 2.397, *p* ≤ 0.04). Parcelling per dark/light period showed the following. In the first dark period, AB and TL larvae were both less active across the time blocks (i.e. showed less *distance moved;* time block: *F*(1,138) = 119.666, *p* ≤ 0.001) with AB larvae less active than TL larvae (strain: *F*(1,138) = 11.686, *p* ≤ 0.001). In the light period, both strains showed increased activity across time blocks (*F*(1,138) = 4.748, *p* ≤ 0.03). In the final dark period both strains showed a decrease in activity across time blocks (*F*(1,138) = 95.020, *p* ≤ 0.001), but AB larvae stronger so than TL larvae (strain × time block: *F*(1,138) = 6.220, *p* ≤ 0.02). From dark to light, both AB and TL larvae decreased activity (time block: *F*(1,138) = 20.566, *p* ≤ 0.001), but TL larvae stronger so than AB larvae (time block × strain: *F*(1,138) = 5.524, *p* ≤ 0.02). From light to dark, both AB and TL larvae increased activity (time block: *F*(1,138) = 29.756, *p* ≤ 0.001).

At the dark-light transition (Fig 1B) AB larvae showed an increase in *maximum velocity*, while TL larvae did not. At the light-dark transition (Fig 1C) AB larvae showed a strong increase in *distance moved*, while TL larvae did not; the values of AB larvae were significantly (*t* = 2.535, df = 138, *p* ≤ 0.02) higher than of TL larvae.

#### Startle behaviour

**Fig 2:**
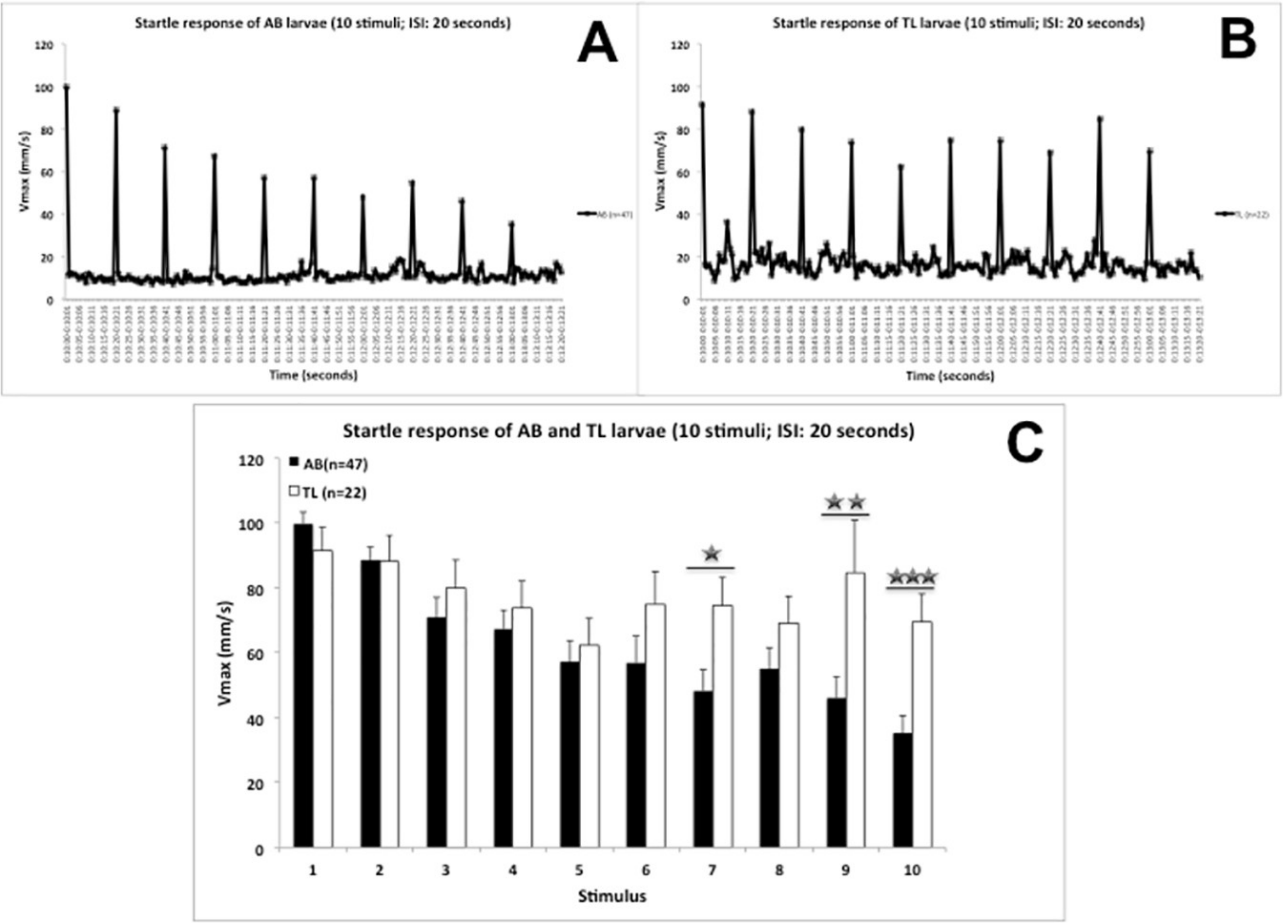
Acoustic/Vibrational startle responses of 5 dpf AB and TL larvae (10 stimuli, 20 s ISI). **Panel A:** Acoustic/Vibrational startle responses of 5 dpf AB larvae. Mean values of *maximum velocity* (Vmax; mm/s) following 10 acoustic/vibrational stimuli and periods without stimuli (10 stimuli; 20s ISI) for AB larvae (N = 1 larva was discarded as it showed no clear response to the first (and subsequent) stimulus (~2 mm/s)). **Panel B:** Acoustic/Vibrational startle responses of 5 dpf TL larvae. Mean values of *maximum velocity* (Vmax; mm/s) following 10 acoustic/vibrational stimuli and periods without stimuli (10 stimuli; 20s ISI) for TL larvae (N = 2 larvae were discarded as they showed no clear response to the first (and subsequent) stimulus (~11 and ~2 mm/s)). **Panel C:** Mean (+SEM) values of *maximum velocity* (Vmax; mm/s) during 10 acoustic/vibrational stimuli with a 20s ISI (peaks of panels A and B) of AB and TL larvae (statistics: between AB and TL larvae: Student’s *t*-test: *: *p* ≤ 0.05, **: *p* ≤ 0.01, ***: *p* ≤ 0.001).

Fig 2 shows the mean startle response to 10 acoustic/vibrational stimuli with a 20 s ISI related to baseline levels (*maximum velocity*: Fig 2A: AB; Fig 2B: TL). It is clear from these graphs that larvae respond to the stimuli (responses clearly above baseline level) and that AB larvae habituate with repeated stimulus presentation more strongly than TL larvae. This is more explicitly shown in Fig 2C, where responses to the 10 stimuli are compared. The data show that AB and TL larvae did not differ in their startle response to the first stimulus, while AB larvae showed a decrease in responding to repeated exposure of the stimulus, while TL larvae did less so, i.e., AB larvae habituated more strongly to repeated exposure than TL larvae. This was confirmed by statistical analysis (two-way ANOVA; factors: strain and stimulus (repeated measure)) showing a significant interaction term: *F*(9,603) = 3.231; *p* ≤ 0.001 (stimulus: *F*(9,603) = 10.137; *p* ≤ 0.001). For stimulus 7, 9 and 10 responses of AB larvae were significantly lower than of TL larvae. To capture strain differences in more detail, strain-wise analyses were done. A highly significant stimulus effect was found for AB larvae (*F*(9,414) = 16.388; *p* ≤ 0.001) in which the response from stimulus 2 onwards was lower than of stimulus 1 (paired *t*-test, *p*-values ≤ 0.05). A significant stimulus effect was found for TL larvae (*F*(9,189) = 1.965; *p* ≤ 0.045) in which the response from stimulus 4 onwards (except for stimulus 6 and 9) were lower than of stimulus 1 (paired *t*-test, *p*-values ≤ 0.05). For both AB larvae and TL larvae values remained above baseline (one-sample *t*-test, *p*-values > 0.05; AB *versus* 10.6 ± 2.2 mm/s (mean ± SD of *n* = 191 time points); TL *versus* 16 ± 4.0 mm/s (mean ± SD of *n* = 191 time points)).

**Fig 3:**
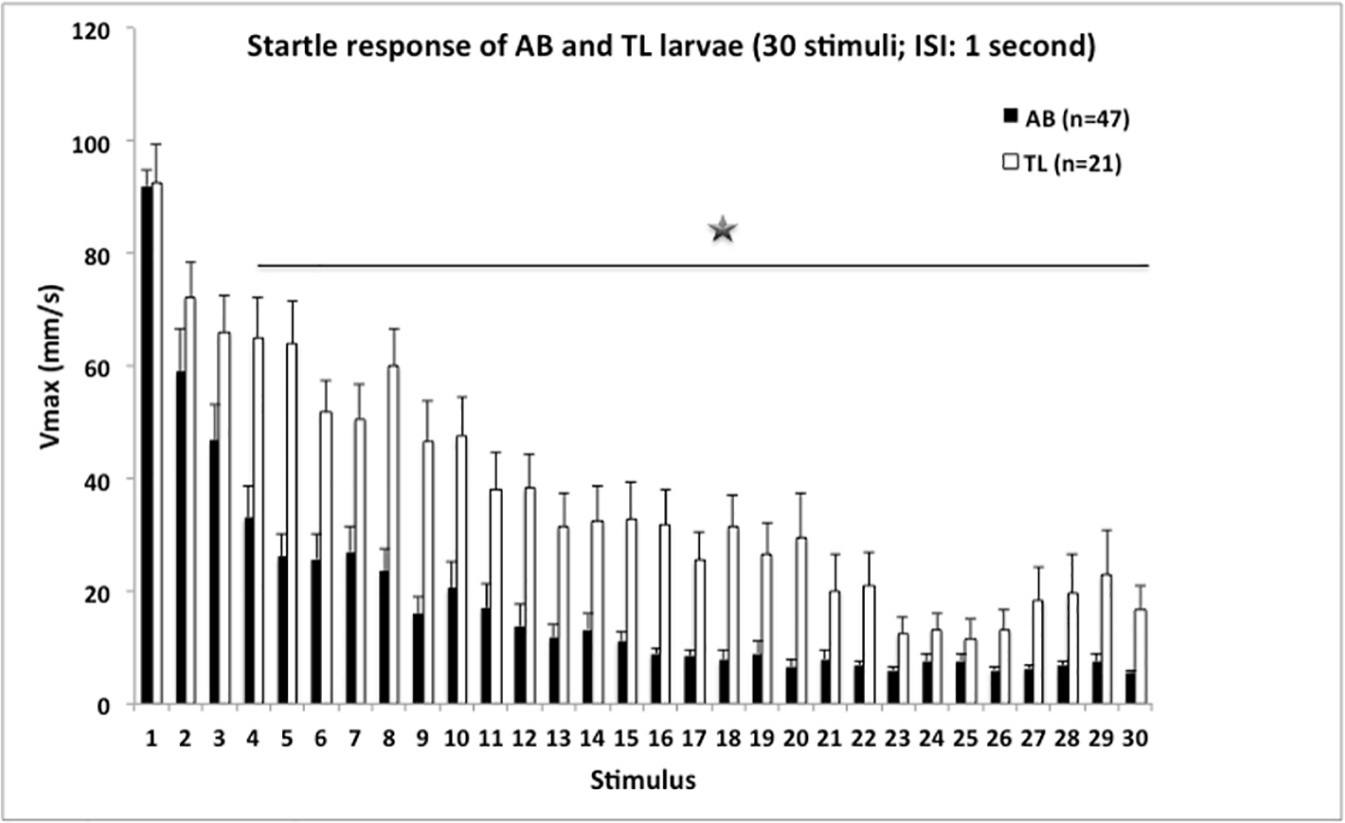
Mean (+SEM) values of *maximum velocity* (Vmax; mm/s) during 30 acoustic/vibrational stimuli with a 1s ISI of AB and TL larvae; statistics: between AB and TL larvae: the black bar indicates that (except for stimulus 25) values of AB and TL differ significantly (Student’s *t*-test, *p*-values ≤ 0.05 or lower (*)). Discarded: N = 1 AB larva and N = 3 TL larvae as they showed no clear response to the first (and subsequent) stimulus (<15mm/s; see also Fig 2A and 2B).

Fig 3 shows the startle response to 30 stimuli with a 1s ISI. The data show that AB and TL larvae did not differ in startle response to the first stimulus, but AB larvae showed a stronger decrease in their response to repeated exposure of the stimulus than TL larvae, i.e. AB larvae habituated faster than TL larvae. This was confirmed by statistical analysis (two-way ANOVA; factors: strain and stimulus (repeated measure)) showing a significant interaction term: *F*(29,1914) = 2.850; *p* ≤ 0.001 (stimulus: *F*(29,1914) = 49.090; *p* ≤ 0.001). From stimulus 4 onwards (except for stimulus 25), AB larvae showed a significantly lower response than TL larvae. To capture strain differences in more detail, further analyses were done strain-wise. A significant stimulus effect was found for AB larvae (*F*(29,1334) = 41.880; *p* ≤ 0.001) and TL larvae (*F*(29,580) = 17.045; *p* ≤ 0.001). The responses of AB larvae were significantly lower compared to the response to stimulus 1 from stimulus 2 onwards (paired *t*-test *p*-values ≤ 0.001); from stimulus 11 onwards responses have reached baseline values (one-sample *t*-test, stimulus 1–10, *p*-values ≤ 0.05). The responses of TL larvae were significantly lower compared to the first stimulus from stimulus 2 onwards (paired *t*-test, *p-values* ≤ 0.05–0.001); from stimulus 17 onwards responses have reached baseline values (one-sample t-test, stimulus 1–16, *p*-values ≤ 0.05).

## Discussion

Our data show that AB and TL larvae differ in baseline HPI-axis activity, expression of (neuro)development related as well as (innate) immune system related genes, light-dark induced changes in motor behaviour as well as habituation to acoustic/vibrational stimuli. Overall, these data confirm our earlier observations in adult fish of the same strains [9] and emphasise that differences between strains need to be taken into account to enhance reproducibility both within, and between, laboratories.

### Gene expression and cortisol

AB larvae showed higher levels of cortisol and expression levels of *crf* and *crfbp* than TL larvae at 5 dpf. These data indicate higher baseline HPI-axis activity in AB larvae than TL larvae and, along with the observation that expression levels of *mr* did not differ between AB and TL larvae, are in line with our earlier findings in adult zebrafish [9]. We did not observe an increase in expression levels of *gr-alpha*; at variance with our study in adult zebrafish. This may be related to the higher expression levels of *gr-beta* in AB larvae than TL larvae. GR-beta has been suggested to act as dominant-negative inhibitor of GR-alpha [47,48]; indeed, when we calculated the *gr-beta*/*gr-alpha* ratio, it was significantly higher in AB larvae than TL larvae (mean ± SEM: 0.17 ± 0.02 (*n* = 10) *versus* 0.010 ± 0.002 (*n* = 10), *t* = 7.04, df = 18, *p* ≤ 0.0001). Whether these data imply that AB and TL larvae differ in stressor induced HPI activity remains to be determined; we are currently addressing this.

We observed increased gene expression levels of a series of markers of (neuro)development in AB larvae compared to TL larvae, *i*.*e*. increased expression of genes related to cell proliferation and/or cell cycle control (*pcna* [23,24]), synaptic formation, neuronal connectivity, survival and migration of new neurons (*bdnf* [24–26]), neuronal determination / differentiation as well as regulation of neuronal survival and maturation (*neurod1* [24,27]), general growth and development (*igf-1* [28,29]) as well as energy homeostasis and neuroendocrine control (*cart4* [32,33]). Collectively these data suggest a better (more extensive) or faster development in AB larvae than TL larvae. The higher expression levels of *gr-beta* in AB larvae than TL larvae may support this, as it was recently suggested that *gr-beta* plays a functional role in larval development [48].

It was recently shown that increased levels of (baseline) cortisol during early embryonic/larval stages increase baseline expression of several (innate) immune system related genes, notably the expression of *irg1l* (*immune-responsive gene 1-like*), the macrophage marker *mpeg1*.*1* and the immune-regulatory gene *socs3a* [15]. In line with this, we observed that in AB larvae, where baseline cortisol levels are higher than in TL larvae, baseline expression levels of *irg1l*, *socs3a*, *mpeg1*.*1* and *mpeg1*.*2* are higher than in TL larvae. The present data thus collectively suggest differences in baseline expression of genes involved in the innate immune response between AB and TL larvae. Whether this leads to differences in responding to infections needs to be studied [34]. Interestingly, Hartig and colleagues [15] have shown that increased baseline levels of cortisol in adult stages are associated with a decreased innate immune response. Whether this would also apply then to AB zebrafish, which have higher baseline levels of cortisol in adulthood than TL zebrafish [9], awaits further study.

### Light-dark behaviour and startle behaviour

We included two behavioural paradigms to study on the one hand the possible functional consequences of differences in baseline HPI-axis activity and neuroplasticity, and on the other to assess whether our strains are comparable to those of other laboratories as differences may occur between laboratories while using the same strain [5,41]. Functionally, the differences in baseline HPI-axis activity and neuroplasticity seem to be expressed in the observations that AB larvae showed (1) a stronger increase in activity in response to changes in light-dark conditions, and (2) a stronger habituation to repeated exposure to an acoustic/vibrational stimulus than TL larvae.

The data from the dark-light-dark paradigm are partly in line with published results. First, activity for both strains was lower in the light period than the dark period [4,17]. Recently, it was shown that increased baseline levels of cortisol (due to microinjections of cortisol into the yolk at the one cell-stage) are associated with increased activity in the light period but not the dark period, of a dark-light paradigm in 4 dpf TL larvae [17]. The observation that activity levels differed in the first dark period (TL higher than AB), but not the light period, suggests that differences here may not be related to differences in baseline HPI-axis activity, unless the finding of Best and colleagues [17] is specific for their procedure or TL larvae. Activity levels in the first dark period (but not the light period) have been shown to be differently related to developmental stage (5–7 dpf) in AB and TL larvae: a decrease in activity with an increase in age in AB larvae with the opposite pattern in TL larvae [4]. In contrast to what we have observed here, in that study AB larvae showed higher activity levels than TL larvae at 5 dpf in the first dark period [4]. To what extent differences in activity in the first dark period between AB and TL reflect subtle differences in developmental stage and reflect laboratory differences between the same strains [5,41] or in procedures needs to be determined. Second, the stronger increase in activity in response to changes in light-dark conditions in AB larvae than TL larvae is in line with results from earlier studies [4,10,11]. Increased activity as a result of sudden dark conditions may (indirectly) relate to activation of the HPI-axis [35–37]. Thus, the higher dark-induced changes in motor activity in AB larvae than TL larvae seems in line with the higher HPI-axis activity in AB larvae than TL larvae.

In the startle paradigm, we observed that the initial startle response did not differ between AB and TL larvae. This suggests no differences in the basal functioning of the Mauthner cells based startle circuitry [49]. AB seemed to habituate to repeated exposure of the acoustic/vibrational stimuli at 20 s inter-stimulus-interval (ISI), while TL did less so. The latter is in line with earlier findings on TL larvae [38,40] and also larvae of the Wild Indian Karotype (WIK) strain [39,40,46]. The strong habituation of AB larvae was therefore surprising and needs further study. The more rapid habituation to repeated exposure of the acoustic/vibrational stimuli using a 1 s ISI in AB larvae than TL larvae, is in line with earlier published data on TL larvae and AB larvae (TL: [38]; AB: [39]). Overall, these data suggest increased habituation or non-associative learning in AB over TL larvae. A recent study in zebrafish larvae showed that IGF1 increases acoustic startle habituation through activation of the IGF1 receptor thereby regulating synaptic strength [40], in line with the proposed role of IGF1 in neuronal plasticity [29]. Hence, we hypothesise here that the difference in habituation between AB and TL may be related to higher levels of expression of *igf1*, that we observed in AB compared to TL larvae. It is clear that this warrants further study. To what extent these strain differences are also related to glutamatergic function involved in startle habituation [38,40,46,49,50] remains to be determined.

### Limitations

This study has a few limitations. First, we sampled only at one time-point. While we observed clear differences between AB and TL larvae in baseline cortisol and gene-expression levels of the HPI-axis, which are similar to what we have observed in the adult stages [9] and differences in the expression of (neuro)development-related genes (as well as (innate) immune system related genes), the possibility should be entertained that we do not look at absolute differences between AB and TL larvae, but rather that TL larvae are lagging behind in development with values being similar at a later time point. This needs further studies. Second, this is a single institute study. It has been shown that genetic and behavioural profiles of the same may differ between labs [5,41]. Hence, our data may reflect local differences, rather than true strain differences. Still, the behavioural data strongly suggest that our AB and TL strains are similar to those of others. A multi-institute study may resolve this issue. Third, and in conjunction with the former, here we studied larvae reared under 14hL:10hD. While this is a rearing condition close to the natural habitat of zebrafish, where embryonic and larval development occur (roughly) under a 14L:10D light-dark regime [51], light regimes used in the past have varied, and in the present still vary, between laboratories, such as continuous dark or continuous light regimes. Continuous dark and continuous light regimes affect hatching rate, increase the occurrence of malformations and lead to different behavioural outcomes in light-dark paradigms compared to 14L:10D [52,53]. Hence, extending the present study to include these conditions may enhance the robustness of findings and allow for characterising these strains in relation to different rearing conditions, such as light regimes. We are currently addressing this.

### Implication and concluding remarks

Earlier, we have observed that adult AB zebrafish compared to TL zebrafish had a higher baseline HPI-axis activity [9]. Increased baseline HPI-axis activity may affect offspring as mothers deposit cortisol and glucocorticoid receptor (GR; both mRNA and protein) in egg yolk, which, among others, have effects on baseline HPI-axis activity, (neuro)development, immune function and motor activity in larvae [14–17]. Overall, our present study clearly suggests that 5 dpf AB and TL larvae differ in levels of gene expression, physiology and behaviour in line with this idea. It should be noted however that the mutated gene (*connexin 41*.*8*) in TL that leads to spots rather than stripes, is also found in the heart, eye and brain [54] and may thus affect development and functioning of these organs. So, the possibility should be entertained that current differences between AB and TL are the consequence of this mutation in TL. Future experiments are directed at the *leopard* strain, which also carries a mutation in *connexin 41*.*8* [54,55] to assess whether similar effects as in TL will be observed. Regardless of the cause of these differences, our data show that strain has an effect on gene expression, physiology and behaviour in 5 dpf larvae, which need be taken into account when designing experiments, as they may have profound effects on reproducibility, both within and between laboratories.
